# Malaria prevention and treatment in migrant agricultural workers in Dangur district, Benishangul-Gumuz, Ethiopia: social and behavioural aspects

**DOI:** 10.1186/s12936-021-03766-3

**Published:** 2021-05-19

**Authors:** Yehualashet Tadesse, Seth R. Irish, Sheleme Chibsa, Sisay Dugassa, Lena M. Lorenz, Asfawesen Gebreyohannes, Hiwot Teka, Hiwot Solomon, Eshetu Gezahegn, Yonas Petros, Mesfin Haile, Mesfin Eshetu, Matthew Murphy

**Affiliations:** 1 USAID|Private Health Sector Project, Abt Associates Inc., Addis Ababa, Ethiopia; 2 The US President’s Malaria Initiative, Bureau for Global Health, Office of Infectious Disease, United States Agency for International Development, 1300 Pennsylvania Ave NW, Washington, DC 20523 USA; 3 Division of Parasitic Diseases and Malaria, Centers for Disease Control and Prevention, 1600 Clifton Road, Atlanta, GA 30329-4027 USA; 4 U.S. Agency for International Development (USAID), Entoto Street, Addis Ababa, Ethiopia; 5 Aklilu Lemma Institute of Pathobiology, Addis Ababa University, Addis Ababa, Ethiopia; 6 Department of Disease Control, London School of Hygiene & Tropical Medicine, Keppel Street, London, WC1E 7HT UK; 7 College of Medicine & Veterinary Medicine, University of Edinburgh, Edinburgh, UK; 8 Disease Prevention and Control Directorate, Federal Ministry of Health, Addis Ababa, Ethiopia

**Keywords:** Migrant worker, Focus group, Semi-structured interview, Malaria, Ethiopia

## Abstract

**Background:**

Sixty percent of the Ethiopia population is at risk of malaria, with the highest prevalence reported in Gambella (6%) and Benishangul-Gumuz (3%) regions. Within these regions are large agricultural developments with high numbers of seasonal migrant workers. The migrant workers are believed to be at increased risk for malaria infection due to their poor living conditions and outdoor activities, but there is little information on their specific behaviours and health risks. This study was conducted to address this gap.

**Methods:**

Quantitative observations were conducted from September to December 2017 in the Benishangul-Gumuz Region. The nightly routines of mobile migrant workers were observed every month for 4 consecutive months. The study team collected quantitative data including nocturnal behavioural observations of worker living conditions, malaria prevention efforts, and work activities and surveys of worker representatives. Qualitative data was collected from migrant workers, farm managers and local health providers using focus group discussions and semi-structured interviews.

**Results:**

Migrant workers arrived in the study area during the peak malaria transmission season and the workers in focus groups reported repeated cases of malaria during their stay on the farms. Overall, less than a quarter of the migrant workers were sleeping under a mosquito net by midnight in all 4 observation months. Some work activities also took place outdoors at night. The study additionally found a lack of access to malaria prevention and treatment at the farms and challenges in utilizing local public health facilities.

**Conclusions:**

There is a need to better address malaria prevention and treatment needs among migrant workers in Ethiopia through outreach from existing healthcare infrastructure and within the farms themselves. This will help prevent malaria transmission both within this population and prevent transmission of malaria back to home communities in lower burden areas in Ethiopia.

## Background

**Fig. 1:**
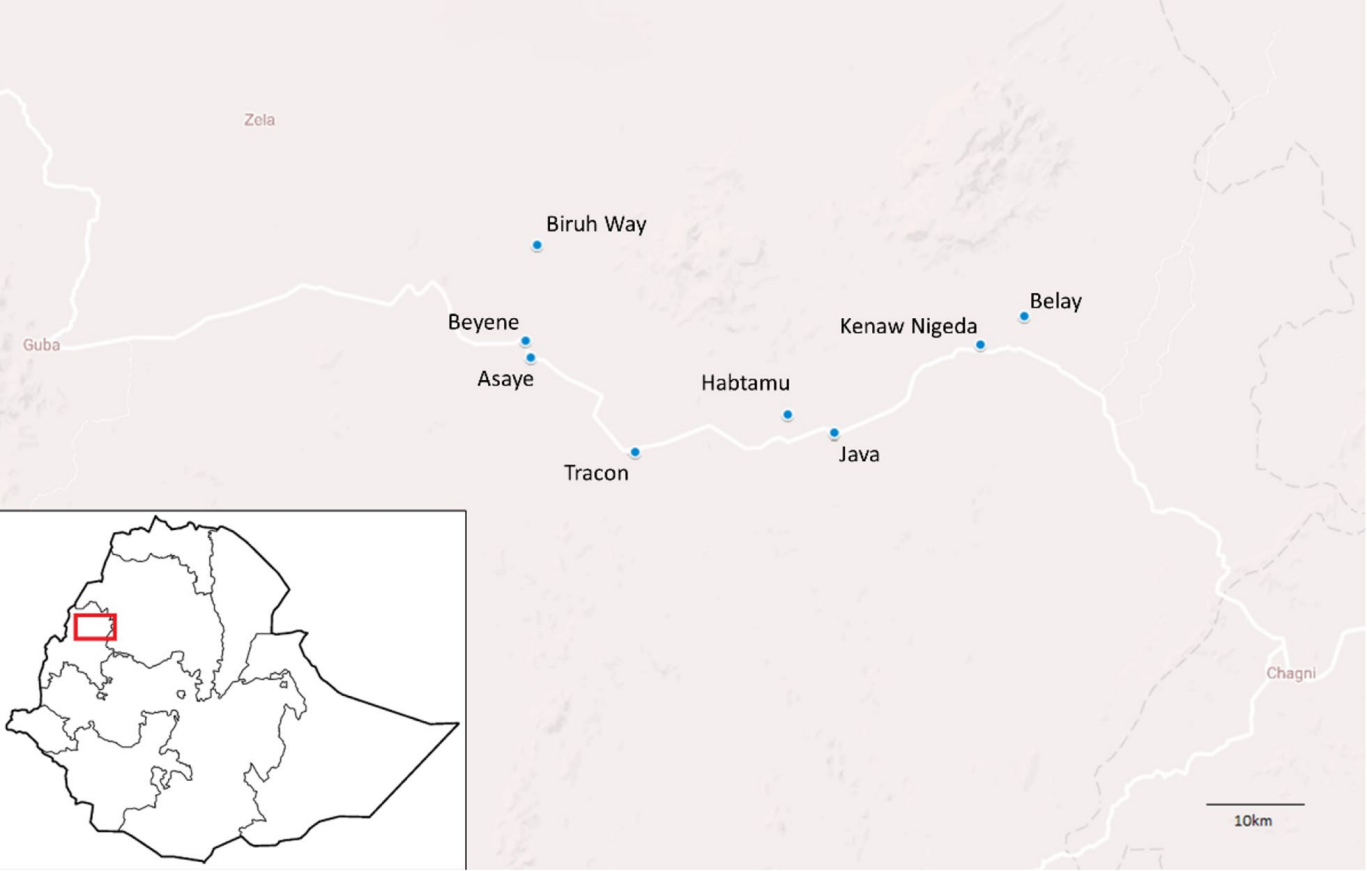
Map of selected farms (blue dots) and location of Dangur district in Ethiopia

Malaria is a life-threatening parasitic disease transmitted to humans through the bite of infected *Anopheles* mosquitoes. The disease is a major public health problem in Ethiopia endemic in large parts of the country with epidemic potential in other areas [1, 2]. A recent transmission risk stratification indicated 60% of the population is at risk of malaria [3], and in 2015, the highest prevalence reported by microscopy was in Gambella (6%) and Benishangul-Gumuz (3%) regions [2]. Benishangul-Gumuz region is where this study was conducted (Fig. 1). Malaria transmission tends to be highly variable geo-spatially within each year as well as between years [2]. In most parts of the country, the peak of malaria transmission follows the long rainy season (July to September) each year. However, some parts of the south, central and east of the country have a short rainy season beginning as early as February which can continue through May [4, 5]. The total number of laboratory-confirmed malaria cases decreased from 2.8 million in 1990 to 1.5 million in 2017 [6, 7]. Simultaneously, a significant reduction in mortality and morbidity has been documented, and central parts of the country are now classified as malaria free or with low transmission [1, 3, 6, 8]. *Plasmodium falciparum* and *Plasmodium vivax* contribute 80% and 8% of malaria cases, respectively (with 12% of results indicating *P. falciparum*/mixed) as determined by rapid diagnostic tests [2]. Some reports indicate recent increases in the numbers of *P. vivax* cases [9, 10]. *Anopheles arabiensis*, a member of *Anopheles gambiae* species complex, is the primary vector of malaria in Ethiopia [11–13].

High population growth, scarcity of farmland in the high and midland areas and the increased investment opportunities in agriculture activities in fertile lowlands of Ethiopia have led to seasonal population mobility into lowland areas in search of work [14]. This population movement has increased exposure to malaria and other vector-borne diseases that are more highly prevalent in lowland areas. With the current increase of population density in malarious lowlands of the country, areas rich with untapped natural resources, malaria will continue to be an important public health problem in the future [15–18]. In addition, the country’s commitment to embark on ambitious mega-projects (especially in lowland areas including sectors, such as agriculture, transport and power) requires movement of enormous numbers of workers to project sites. The current national malaria strategic plan aims to maintain near zero malaria deaths, reduce malaria cases by 40% (from the 2016 baseline) and eliminate malaria from Ethiopia by 2030. Malaria among mobile and migrant labour will undoubtedly challenge the realization of this aim [3].

Labour movement has historically impacted malaria control and elimination efforts. For instance, in Swaziland, malaria was eliminated in the 1950s through successfully implemented control measures. However, agricultural developments that attracted a migrant labour workforce from endemic Mozambique led to the resurgence of malaria [19]. Mobile and migrant workers are also among the key challenges for malaria elimination and contribute to the spread of drug-resistant parasites in the Greater Mekong Sub-region [20, 21].

Travel to malarious areas has also long been recognized as a risk factor for malaria infection [22–24]. The population movement can increase malaria transmission through exposure of non-immune populations to malaria, poor living conditions in new settlements (compared to the settled populations), high risk work activities (outdoors at night time which increases contact with mosquitoes), and malaria control policies that do not effectively address migrant worker needs [25]. Mobile and migrant workers may serve as carriers who transport parasites to the highlands and other locations in Ethiopia with lower malaria prevalence [26].

The behaviour and living conditions of migrant workers are major determinants of their vulnerability to mosquito bites. Gryseels et al*.* [27] demonstrated heterogeneity of human behaviour in the forested region of Cambodia and included risk behaviours such as commuting between farms in the forest (where mosquito bite exposure is high), varied sleeping times due to economic activity, and irregularities in the use of long-lasting insecticidal nets (LLINs) [28].

In Ethiopia, the relationship between the risk of malaria experienced by migrant workers and their behavioural and socio-cultural context is poorly understood. This study aimed to describe the behavioural factors and living conditions of migrant workers in relation to their risks of malaria to inform future control and elimination efforts.

## Methods

### Study design

A mixed-method study using quantitative and observational methods was conducted from September to December 2017 in Dangur District. Eight farms were selected, with two shelters selected per farm for a total of 16 shelters. Migrant workers were observed once monthly for 4 consecutive months. The study team collected quantitative data by: (1) nocturnal observation of migrant workers in their shelters; (2) nocturnal observation of a group of migrant workers while they worked in the fields; and (3) surveys of migrant workers in shelters at the beginning and end of the observational period (Table 1). Qualitative data was collected using focus group discussions (FGDs) and semi-structured interviews (SSIs) with migrant workers, farm managers and health workers.

**Table 1:** Summary of data collected each month in Dangur district, Benishangul-Gumuz region, Ethiopia

Month	# of pre-post observation surveys	# of worker shelters observed	# of field observations	# of FGDs	# of SSIs
September	16	16	2	0	0
October	16	16	7	12	0
November	16	16	6	0	32
December	14	14	4	0	24
Total	62	62	19	12	56

### Study site and sampling

**Fig. 2:**
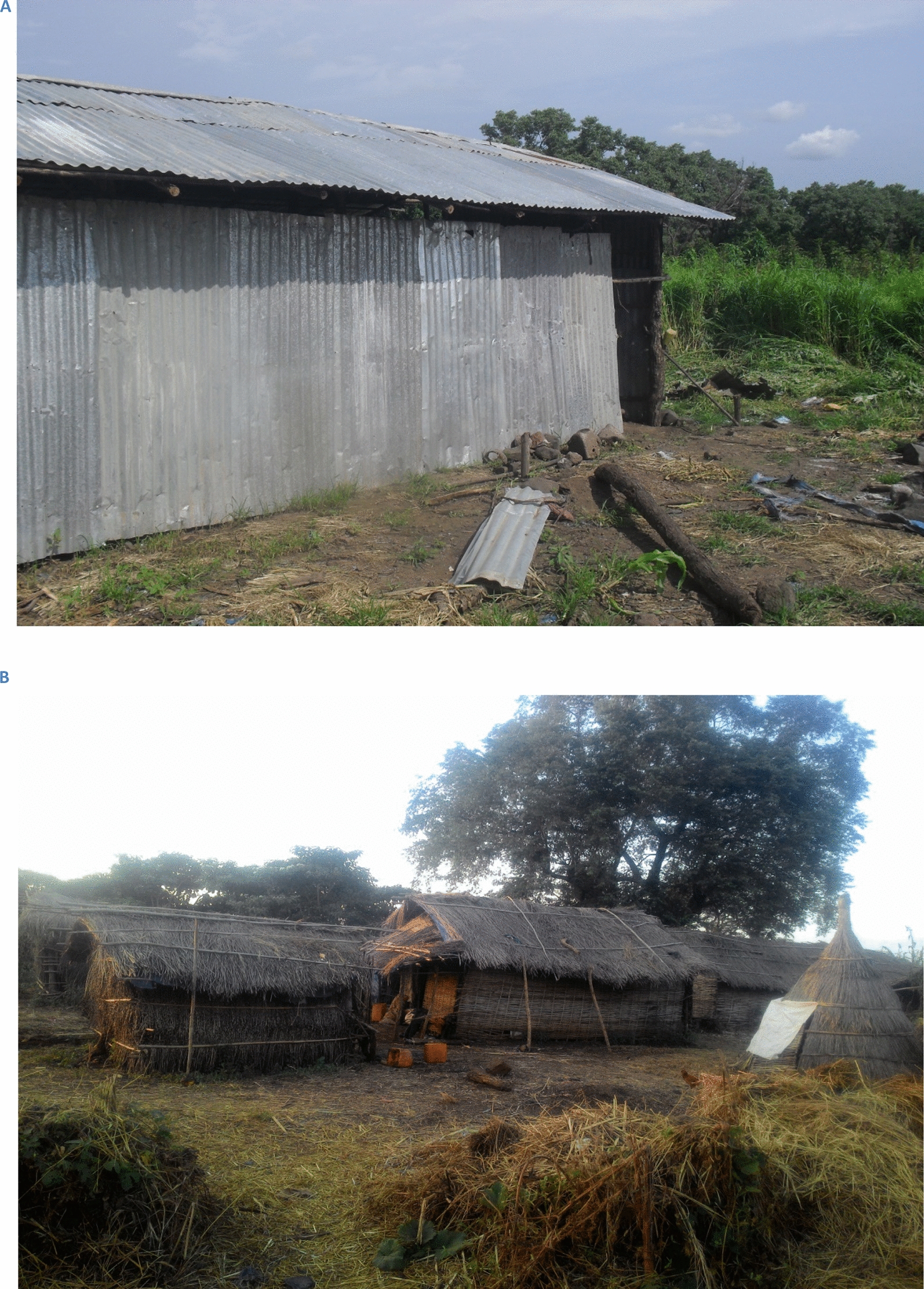
Seasonal migrant workers stay in temporary shelters on agricultural farms. Typical shelters are made from a variety of materials including iron sheeting (**A**) and grass (**B**)

Dangur District is located in Metekel Zone and is found in the northern part of Benishangul-Gumuz Region in western Ethiopia (Fig. 1). The climate of the area alternates between a rainy season (June to September) and a long, dry season (October to May). The mean annual rainfall in the district ranges from 900 to 1400 mm per year. The main economic activity of the native population (which includes Gumuz and Shinasha ethnicities) is mixed farming involving both growing crops and raising of livestock. Large numbers of agricultural labourers migrate to the district, especially from neighbouring Amhara region, to work in the commercial farms during the farming season, which coincides with the rainy malaria transmission season. Most migrant workers stay in large temporary communal shelters made of wooden frames covered with grass (Fig. 2). Others stay in shelters made of wooden frames covered with corrugated iron sheets. Migrant worker shelters visited in this study were temporary structures where migrant workers rest and sleep after work. The elevation of migrant worker shelters ranged from 774 to 1183 m (above sea level) with an average of 936 m.

After enumerating all the established commercial farms in the district, four large-scale farms (larger than 100 ha) and four small-scale farms (less than 100 ha) were selected in collaboration with district officials. The farms were selected taking into account multiple factors including their accessibility, presence of migrant workers (or at least planned employment for the current season) and agreement of the farm management to take part in the study. Two shelters with the largest number of inhabitants were selected per farm making a total of 16 shelters. Nocturnal observation of inhabitants of shelters, field observation, pre and post-observation surveys of shelters were conducted in all the farms and shelters. All the 16 shelters were reached once every month. Any worker who was there at time of observation was observed (Table 1).

### Standardized migrant worker shelter survey

A standard questionnaire was administered to a representative from each migrant worker shelter the night prior to human observation during each month from September to December 2017 (Table 1). These workers who were knowledgeable about the farm shelters and represented the shelters for farm administrative duties were also invited to respond to questions related to migrant worker shelters. The representative provided information on the number of people staying in each shelter, their age, gender, and pregnancy status if female. Characteristics of the shelter, such as building materials and availability of LLINs for those sleeping in the shelter were recorded. The location and elevation of each shelter was recorded using a GARMIN GPSMAP® 64st (Garmin International Inc., Olathe, Kansas, USA).

Each month, a follow-up questionnaire was administered to the shelter representative about his/her behaviour and net use the previous night, including the time he/she went to bed, whether he/she used an LLINs, what time he/she woke up and methods used to prevent mosquito bites.

### Human observation

Human behaviour was observed by trained research assistants from 1700 until 0900 the following morning. A total of 16 worker shelter observations were completed each month between September and December. A total of 19 worker observations in the field were completed in 4 months. All workers were enumerated and given a two-digit ID to anonymize them. One observer recorded the worker behaviour from 17:00 to midnight, and the other observer recorded from midnight until 9:00. Indoor or outdoor location, net use and activities of each shelter member were documented every thirty minutes until midnight and then every hour afterwards.

### Focus groups

A total of twelve FGDs were conducted in October 2017. Participants were recruited from large-scale farms (5 FGDs), small-scale farms (5 FGDs) and Mambuk, a town where worker recruitment is common (2 FGDs). The number of participants for each FGD ranged from 8 to 12 individuals. Eight FGDs were composed of only male migrant workers, one group consisted of only female migrant workers and wives of migrant workers, and three FGDs were a mix of both male and female participants.

An FGD guide was used to provide consistency between groups, and discussion topics included typical working and sleeping hours, temporary settlements and shelters, sleeping habits and indoor and outdoor activities. Malaria treatment options and practices were also discussed. In addition, the discussion explored barriers and facilitators to the use of malaria prevention interventions in the context of migrant workers and risk perceptions of contracting malaria.

### Semi-structured interviews

Semi-structured interviews were administered to farm managers, migrant workers, and health workers in nearby health posts and health centres. The interview guide was developed specifically for each category and was modified to include key issues that arose during the FGDs. The guide focused on understanding migrant workers’ behaviour in relation to malaria prevention and treatment. The interview addressed topics such as general profile of the interviewee, perceived exposure to malaria, common illnesses in the district, knowledge of malaria prevention, attitude towards use of prevention interventions, challenges and recommendations related to malaria prevention and treatment options.

### Data analysis

Quantitative data was double entered using SPSS Statistics 21.0 (SPSS Inc., Chicago, USA). Any discrepancy between the two data sets was corrected by referring to the paper form. Data were analysed using STATA 11.0 (Stata Corp, College Station, USA) and were organized and summarized using descriptive statistics.

Qualitative data were analysed using a framework analysis. The research team transcribed and translated each interview and focus group discussion. The translated material was reviewed multiple times to obtain a general understanding of the content. A thematic approach was followed during the analysis. It included a descriptive phase of identifying meaning units and assigning codes which were then compared and reorganized into tentative frames (or themes). NVIVO 11 (QSR International Pty Ltd. Cardigan UK) was used to assist this process.

## Results

### Survey of worker shelters

The monthly average number of workers in each shelter ranged between six and ten. The majority (> 80%) of workers were males and most of them (> 90%) had stayed in the shelter the previous night. At the time of the study, none of the female workers reported being pregnant. The majority of the workers had either no education or only a primary level of education and this pattern was similar for each month.

The study team did not witness construction of new temporary worker shelters at the selected farms. Migrant workers stayed in temporary shelters established in previous years so there was no monthly variation of shelter construction. The roof of most (63%) shelters was grass while walls were made of grass, iron sheeting, plastic sheeting or bamboo (> 90%). Most shelters had open eaves (94%). Surface water was the main source (75%) of drinking water (Table 2).

**Table 2:** Characteristics of migrant worker shelters (N = 16)

Shelter characteristics	Number (%)
Material of roof:	
Grass	10 (63)
Thatch	1 (6)
Iron sheet	4 (25)
Other	1 (6)
Eaves:	
Open	15 (94)
Closed	1 (6)
Material of wall:	
Grass	7 (44)
Plastered	1 (6)
Other (Bamboo, iron sheet, plastic)	8 (50)
Source of drinking water:	
Surface water	12 (75)
Protected well	1 (6)
Hand dug well	3 (19)

None of the shelters had window screening and all the floors of the shelters were made of mud. The number of rooms per shelter ranged from two to eight, but varied slightly between months due to modifications made to accommodate influx and outflux of migrant workers. Almost all rooms in a shelter were used for sleeping. The mean number of sleeping places per shelter was three and ranged from two to 11. No shelters had latrines, and firewood was used for cooking in all locations. All shelters had at least one individual with a mobile phone. Only two shelters had individuals with their own transportation (a donkey and a truck).

**Fig. 3:**
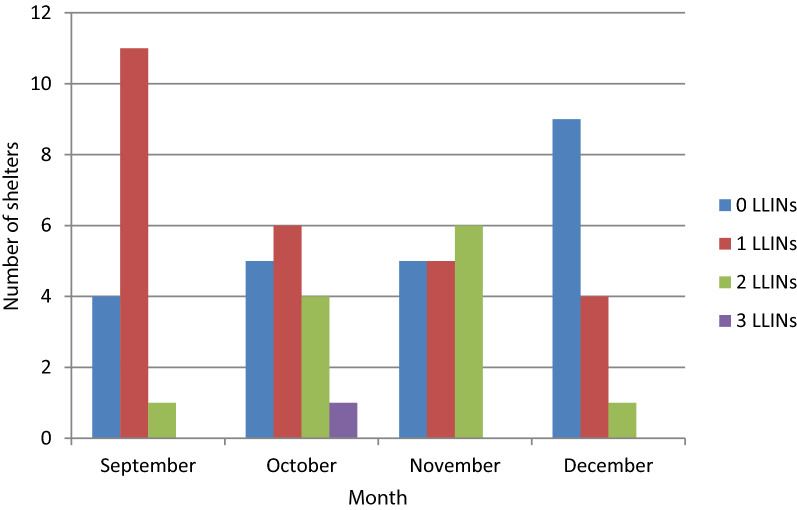
Availability of mosquito nets (LLINs = Long-Lasting Insecticidal Nets) in migrant worker shelters indicating the presence of 0 to 3 mosquito nets per shelter each month. Length of bars indicate the number of migrant worker shelters with the specified number of mosquito nets

Out of 16 shelters, 12 (75%) owned at least one mosquito net in September (Fig. 3). There was one mosquito net for every 7 to 14 migrant workers in each shelter, depending on the month. Three-quarters of the bed nets available in September were less than 36 months old (Table 3). Most of the bed nets were hung above elevated sleeping benches made from long poles. Only blue, rectangular bed nets were found in the shelters, with PermaNet as the dominant brand. Most bed nets had been obtained through a health facility or community agent free of charge. All bed nets were used by someone the previous night and the majority were shared by two or three people. No distinction was noted among farm types (large and small) and this applies to all sections of the result.

**Table 3:** Characteristics of available bed nets including number of migrant workers per net

Characteristic of bed net	Number of net (%)			
	September	October	November	December
Number of workers for each net:	9	7	10	14
Months since obtained:				
≤ 36	10 (77)	14 (88)	10 (59)	1 (17)
> 36	3 (23)	2 (12)	7 (41)	5 (83)
Sleeping place where net used:				
Bed frame (finished)	2 (15)	0 (0)	1 (6)	0 (0)
Bed frame (sticks)	11 (85)	16 (94)	14 (82)	6 (100)
Ground	0 (0)	1 (6)	0	0 (0)
Reed mat	0 (0)	0	2 (12)	0 (0)
Brand of net:				
PermaNet	12 (92)	16 (94)	16 (94)	6 (100)
MAGnet	1 (8)	1 (6)	1 (6)	0 (0)
Source of net:				
Health facility	4 (31)	4 (24)	6 (35)	1 (17)
Community agent	4 (31)	6 (35)	6 (35)	3 (50)
Family or friends	1 (8)	4 (24)	2 (12)	0 (0)
Shop or supermarket	2 (14)	2 (11)	0 (0)	0 (0)
Market	1 (8)	1 (6)	3 (18)	2 (33)
Other	1 (8)	0 (0)	0 (0)	0 (0)
Paid money for net:				
Yes	3 (23)	4 (24)	3 (18)	2 (33)
No	10 (77)	13 (76)	14 (82)	4 (67)
Don’t know	0 (0)	0 (0)	0 (0)	0 (0)
Net location at time of interview:				
Hanging loose over bed/mattress	10 (77)	12 (71)	11 (64)	5 (83)
Hanging and folded up or tied	3 (23)	5 (29)	3 (18)	1 (17)
Not hanging but not stored	0 (0)	0 (0)	2 (12)	0 (0)
Stored away unpacked	0 (0)	0 (0)	1 (6)	0 (0)
Someone slept under the net the previous night:				
Yes	13 (100)	17 (100)	17 (100)	6 (100)
No	0 (0)	0 (0)	0 (0)	0 (0)
Number of individuals using the net the previous night:				
1	4 (31)	7 (42)	6 (35)	2 (33)
2	4 (31)	5 (29)	8 (47)	0 (0)
3	5 (38)	5 (29)	3 (18)	4 (67)

### Human behaviour observations in the worker shelters

**Fig. 4:**
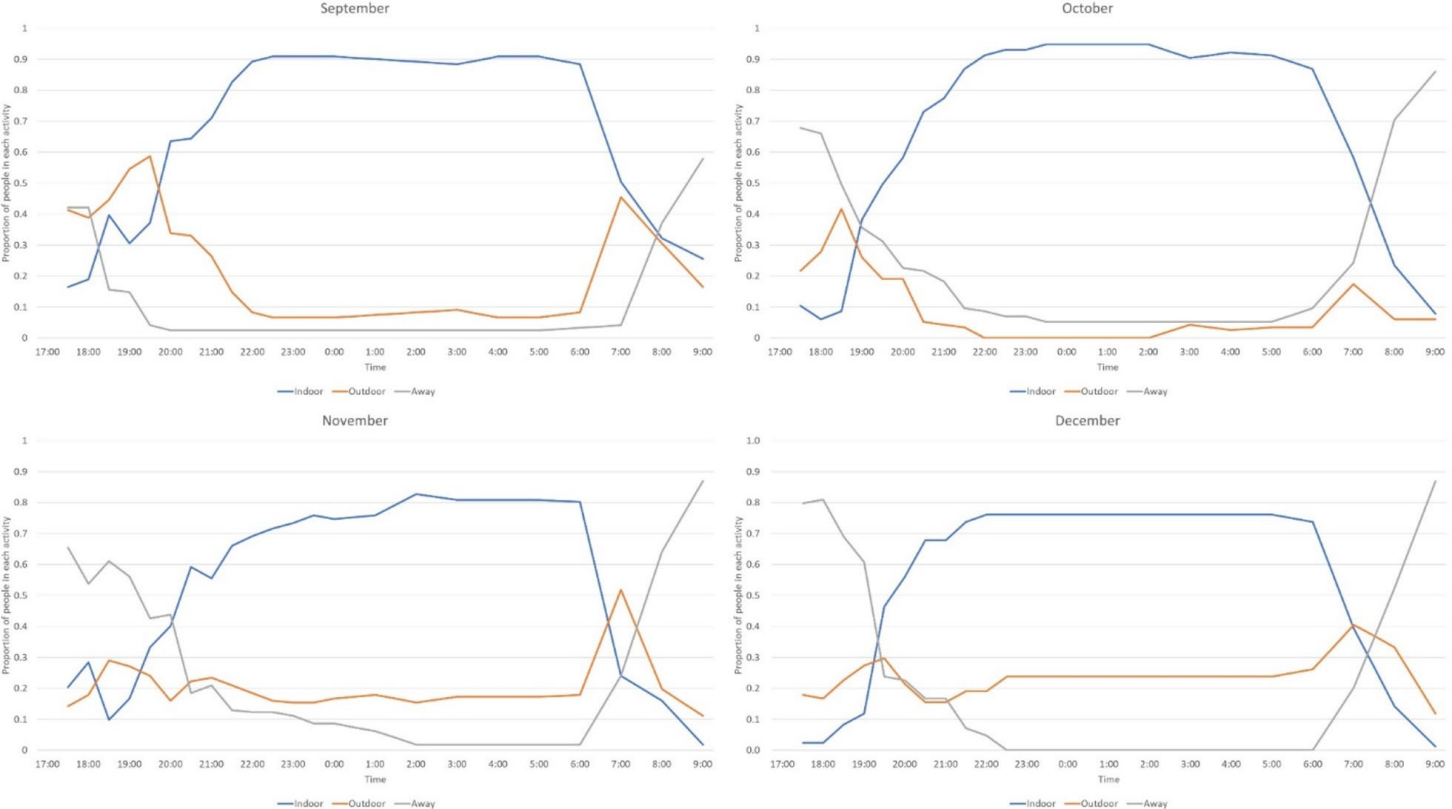
Temporal patterns of human movement indoors and outdoors around temporary shelters for agricultural migrant workers show movement of workers indoors by 20:00, and most were indoors by 22:00

The number of migrant workers observed each month ranged from 162 in November to 84 in December. From approximately 20:00, there were always more people already indoors compared to outdoors or compared to those away from the shelter (Fig. 4). Nearly 50% were already outdoors or away from the shelter by 7:00 the next morning in September, which increased to 75% in November. The percentage of workers away from the shelter increased in later months compared to September (Fig. 4). During November and December, approximately 20% of workers remained outdoors during the whole night. Overall, an average of 87% of workers were indoors at midnight in all 4 months of observation.

**Fig. 5:**
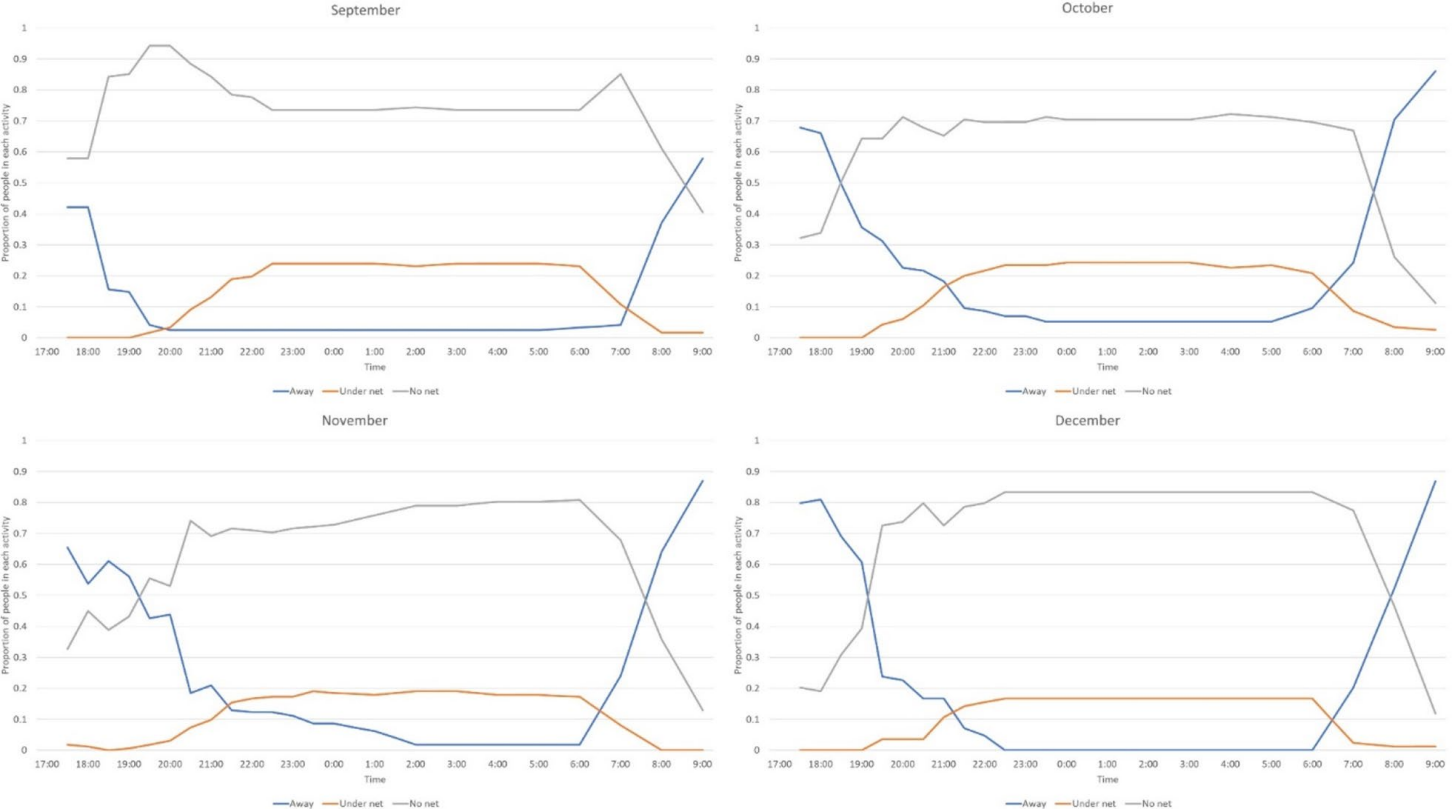
Temporal patterns of mosquito net use among migrant workers indicating less than or close to a quarter of migrant workers were using mosquito nets during each month. The red line indicates the proportion of migrant workers using a mosquito net

More than 70% of migrant workers were not using bed nets during the observations (Fig. 5).

The highest percentage of workers recorded sleeping under a net at any one time was 24%. Those with access to bed nets began using the bed net from 20:00 at night and the majority of those with nets were underneath a bed net by 22:00. The workers had all left the nets by 7:00 the next morning with the majority leaving the protection of the net at 6:00. This pattern was similar across all 4 months (Fig. 5). No worker was observed using other mosquito prevention interventions except for three individuals who tried to repel mosquitoes in the evening using smoke from firewood.

**Fig. 6:**
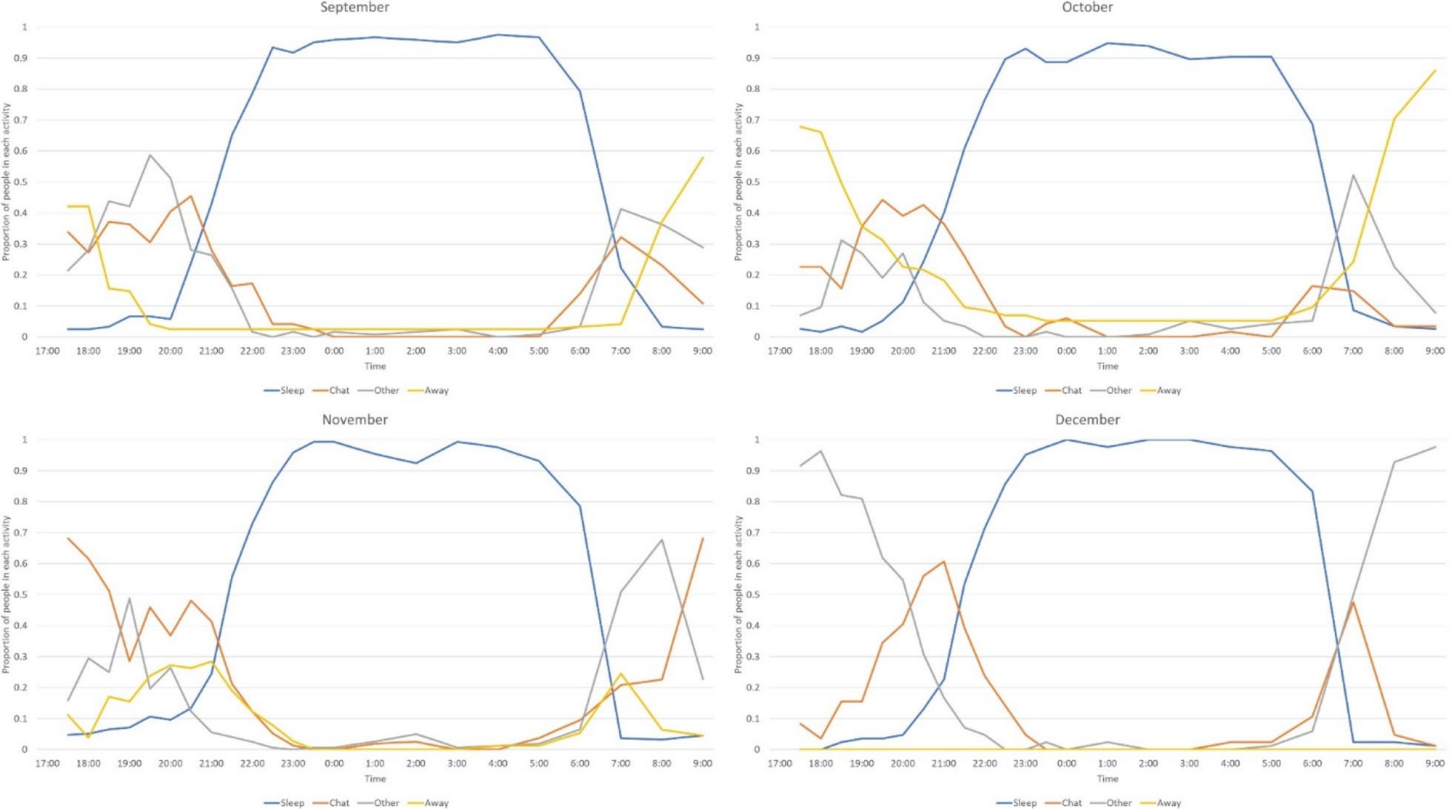
Temporal patterns of activity of migrant workers. Major observed activities of migrant workers after 17:00 included sleeping and chatting

The most common activities among migrant workers after 17:00 were chatting, sleeping, cooking and eating. Up to 40% of workers were still away from the shelters until 20.00 (Fig. 6). Though almost all workers were sleeping by 22:30 in September and October, 10% of migrant workers were chatting or away from the shelter at 22:30 in November and December (Fig. 6). Overall, an average of 98% of migrant workers were sleeping at midnight and 98% were already awake at 08:00 the next morning during the 4 months of observation.

### Human behaviour observations in the fields

The most common field activity observed between 17:00 and 9:00 was harvesting (63%). Other field activities included caring for animals and storing harvest. The average number of migrant workers conducting field activities during the night was seven and ranged from two to 24. The duration of field activities throughout the study ranged from three hours to 16 h with an average of 14 h. Nighttime field activities started at 17:00 to 19:00 (usually at 17:00) and ended the next morning (8:00 to 9:00).

Almost all migrant workers who were working in the field were male adults. They wore shorts and short-sleeved shirts. They had sandals (“ergendo’’) as footwear and covered their head with a scarf. They wore head torches at night. None of the observed workers used any personal protection measures against mosquitoes while in the field.

### Results of post observation survey

Sixteen shelter representatives responded to the survey following the night observations in September (Table 4), and the survey results were consistent across all months. Half of the respondents slept under nets and reported going to bed at 21:00 the previous night. The majority reported that they slept indoors with half of respondents inside by 20:00. Almost none of the representatives slept outdoors in any of the months. None of the representatives used preventive measures to prevent mosquito bites between 18:00 and 20:00, but three wore long-sleeved shirts after 20:00 to prevent mosquito bites in September.

**Table 4:** Migrant worker behaviour indicators collected during the post observation survey for the month of September

Survey respondent behaviour indicators	Number (%)
Slept under net last night:	
Yes	7 (44)
No	9 (56)
Time went to bed previous night (hour):	
20:00	3 (19)
21:00	8 (50)
22:00	4 (25)
23:00	1 (6)
Time woke up in morning (hour):	
01:00	1 (6)
05:00	2 (13)
06:00	5 (31)
07:00	8 (50)
Slept indoor/outdoor:	
Indoor	15 (94)
Outdoor	1 (6)
Location between 18:00 and 20:00:	
Primarily inside	8 (50)
Primarily outside	8 (50)
Activities between 18:00 and 20:00:	
Eating	8 (50)
Chatting	7 (43)
Cooking	1 (7)
Activities between 20:00 and the time you went to bed:	
Chatting	6 (38)
Eating	6 (38)
Sleeping	1 (6)
Drinking alcohol	2 (12)
Watching TV	1 (6)
Bite prevention attempts between 20:00 and time worker went to bed:	
Wore long sleeves/pants	3 (19)
None	13 (81)
Location between the time you woke up and 07:00:	
Primarily inside	10 (63)
Primarily outside	5 (31)
Away from home	1 (6)
Activities between the time you woke up and 07:00:	
Taking bath	2 (12)
Chatting	5 (31)
Washing clothes	1 (6)
Eating	2 (13)
Going to church and others	6 (38)
Bite prevention activities between the time woke up and 07:00:	
Wore long sleeves/pants	3 (19)
Used a net	2 (12)
None	11 (69)

### Qualitative interviews

Almost all the workers responding to interviews came from West Gojam, East Gojam, Awi zone and South Gondar zones of Amhara region. Most arrived at the farms in June and returned home by December, although some reported they may stay later than December to harvest peanut and cotton. However, all respondents agreed that the conditions would be unfavorable for work after December when the weather becomes hotter and rivers dry out, making it difficult to obtain drinking water.

Workers find employment in two ways: either through an agent or on their own. Workers contact agents in their hometown or they travel to the small towns near the farms to arrange work with the agents. Recruitment through an agent is often based on prior written agreements between the agent and the farm employers, which help to safeguard the workers’ rights and benefits (in contrast to those that are self-employed). Those who search for work on their own travel directly to the farms or congregate at specific locations in the nearby towns waiting for employers to request labour. Only a few workers reported having a family back home and none brought families to the farms. All agreed that the living conditions at the farms were not suitable to accommodate a family.

#### Living conditions of migrant workers

All workers reported staying in large, temporary communal shelters which were constructed by wooden frames covered with grass or iron sheeting (Fig. 2). The shelters have no doors, so the structures always remain open to the outside. Most workers try to cover the floor with plastic sheets to sleep while some workers prepared a bedding area to raise themselves above the ground to gain protection from snakes and scorpions. Most farms have a separate dedicated housing area for cooking. During peak times, the respondents reported that the number of inhabitants per worker shelter could exceed 100 workers each night. Sleeping spaces are distributed on a “first-come first-served basis’’. Workers could be forced to sleep outside when shelter gets full, especially during the hot season. Some workers prefer to stay away from the shelters and sleep in the fields. One participant explained his reason to avoid sleeping in the shelters as follows:

“… I sleep on the field. It is smelly inside. I enter the temporary shelter when it rains.’’

Most respondents worked every day of the week except Sunday and religious holidays. Working hours usually started at 07:00 and ended at 18:00, though some reported working at night (especially during harvesting). Work at night does not usually continue past midnight though some of the workers sleep in the field after nighttime work.

The farm workers were divided into two groups: temporary and permanent. Temporary workers (who were the majority) were either paid daily (fixed amount in a so-called labour scheme) or paid per hectare of weeding or harvesting (called a contract scheme). There were also groups of workers who arranged with farm owners (especially in small farms) to share in the profits from the crops. One worker explained a typical day at the farm as follows:

*“… When you do contract work (payment per hectare), you will have your own schedule. When you work as a labourer, you will have a fixed schedule. However, all migrant workers wake up in the morning (at 06:00), work till midday, rest for lunch and then work starting from 14:00 to 18:00. Some contract workers work in the night to avoid the overhead sun. We go to sleep between 22:00 and 23:00.’’*

Sleeping times were variable. However, most workers reported going to sleep between 21:00 and 23:00. Most said they sleep earlier during times of harvesting, as they are aiming to arrive early to their worksite the following morning. The type of produce could also affect sleeping schedule. One worker said:

*“… During sesame harvesting, I sleep during the daytime and work at night. Sesame harvesting is done at night (you may get sick if you do it on sunny days and the seeds will fall off too). I work in the night till 03:00 and then go to sleep. We wake up at 10:00 and start cooking our food. During other times, we go to sleep between 20:00 and 21:00. We wake up at 06:00. Lunch time is from 12:00 to 14:00.’’*

All workers agreed that they had better living conditions in their home towns, but that they have to accept the difficult conditions working at the farms to earn money and improve their lives. However, some mentioned that they could not achieve their goals. The cost of living (including medical costs) was high on the farms, limiting their ability to save money.

#### Perceptions about malaria and its occurrence among migrant workers

**Fig. 7:**
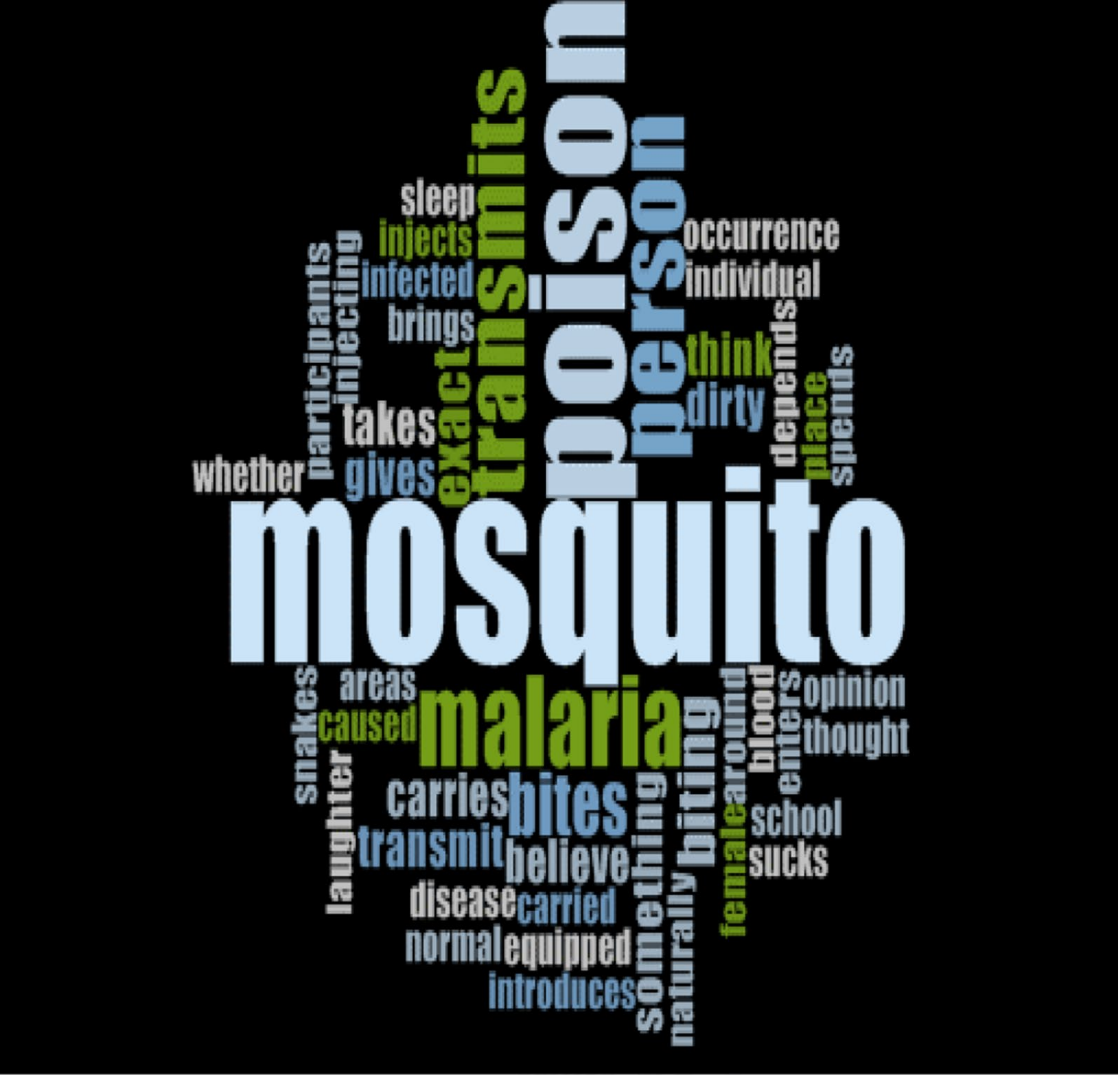
Word cloud for the cause and the transmission of malaria as explained by migrant workers (NVIVO 11)

Most workers believe that malaria is caused by a poison from mosquitoes and that the mosquito transfers the poison from person to person (Fig. 7). Some workers also explained how the mosquito has naturally occurring poison similar to snakes. One worker described it as follows:

“… The mosquito bites us and it introduces its poison into our body.’’

Exposure to cold, lack of food and poor personal hygiene were also reported as the other causes of malaria by workers. All workers assumed that they were at risk for malaria while employed on the farm. One worker elaborated about the risk of malaria while in the farms as follows:

“… The risk is higher here. We take the malaria from here to our hometown. There is no malaria in our hometown.’’

Many workers reported several incidences of malaria in recent months and some of them were on anti-malarial medication during the interview. Almost all workers reported febrile illness after arrival and they considered their hometowns to be safer locations. Some workers also said illnesses were more common among newcomers. All respondents agreed that malaria was the most common diagnosis among the migrant worker population, followed by diarrheal diseases.

#### Knowledge on malaria prevention and availability of prevention kits

Most workers agreed that it was possible to avoid becoming sick from malaria while working on the farms. Using bed nets was mentioned as an important prevention tool and many hoped that obtaining one would bring some relief from the repeated attacks of fever and headache from malaria. Some workers described the malaria-free intervals they had enjoyed while using bed nets in the past (previous years or in other locations). They also pointed to the importance of other prevention measures such as eating good food, drinking clean water, keeping good personal hygiene and environmental hygiene (which was defined as draining swamps, cutting the grass or removing garbage) and prompt medical treatment. Other rarely mentioned prevention methods included the use of smoke from firewood and farm chemicals which were reported to help chase away the mosquitoes.

Few informants reported owning a bed net and none of the farms provided bed nets to workers. The workers were not allowed to get bed nets from the local health posts which required people to show identification to prove that they were from the local area. All respondents agreed that the only option for obtaining bed nets was by purchasing them from local drug shops or from local community members. Prices to purchase bed nets ranged from 40 to 80 ETB ($1.5–$3 USD) and purchasing bed nets usually involved traveling to nearby towns. In contrast, respondents said they had free bed nets provided at their original homes. The reason why they travel to the farms without their bed nets is that they usually share their bed nets with family members and did not want to leave them unprotected.

Most workers complained that they were never given a bed net while working on the farms. The few bed nets owned by some of the workers were old and torn and were either bought or borrowed from a friend. One worker explained his account as follows:

“… I have a bed net but it is old. It has holes and doesn’t kill the mosquitoes when they land. I got it when I was working for an Indian company.’’

*“…We can’t get bed nets here. They don’t give a bed net for migrants. They needed a local ID. We have been asking the health extension workers in my previous farm but didn’t succeed.’’*

Respondents said they tried to cover their whole body with their cloth while sleeping to protect from the mosquitoes and the nuisance of biting insects which is more intense during and immediately following the rainy season. However, they still find their legs, hands and face covered with bites in the morning.

Farm managers and health workers did not deny that there was low utilization of bed nets at the farms, mostly due to lack of access. They confirmed that bed nets have never been distributed to migrant workers except when the district health administration rarely supplied bed nets to the farms based on a request from the farm managers. The health workers said the provision of community health and malaria prevention services were household-based and that this has contributed to the exclusion of the migrant population. In addition, many migrant workers were working far from community health services, making it logistically challenging to reach them.

#### Getting prompt and proper treatment

Most workers agreed that malaria can be treated, although some workers seemed ambivalent. Their doubt was caused due to repeated malaria illness despite their attempt to find treatment in the past. Some workers tried to explain their stance as follows:

“… It can be treated. But there is no complete cure. The treatment is temporary. The malaria will come again as long as we are here in the farm.’’

Fever and malaria illness are overwhelmingly common. Almost all respondents reported at least one malaria illness per month. Most workers knew the symptoms of malaria and seek prompt care though none of the farms have clinics for immediate care and treatment. When workers contracted fever they travelled to the nearby health centres and health posts (which could be up to 30 km away). Some farms occasionally arranged transport for ill workers, but other farms did not provide any transport and the ill workers needed to use public transport. Less commonly, some workers procured their own anti-malarial drugs (mostly Coartem®). One worker explained his routines after fever as follows:

*“…I have Coartem in my bag. When I feel the symptoms, I take it. I buy it from a local pharmacy or borrow from a friend. I mostly take the first four tablets, if symptoms improve, I stop. If I don’t improve, I take the next dose. If there is no change after this, I go to the nearby health facility. Most of the time, the first dose improves the symptoms. However, I usually fail to complete the whole treatment and the symptoms return after a week.’’*

Some farms stock anti-malarials (mostly Coartem) which they sell to workers without using a diagnostic test (price: 50–70 ETB/$1.8–$2.6 USD). Workers sharing anti-malarials among themselves (or keeping some tablets for future illness) was a common practice. It was rare to find a worker using a traditional medicine for malaria and fever treatment. However, one worker did report chewing a bitter plant (locally called “neem’’) when he could not afford to buy Coartem. He preferred Coartem to “neem” [referring to leaves of *Azadirachta indica*], but did say that “neem’’ was helpful.

Drug stock-outs were commonly reported in the local health posts and health centres near the study area. As previously mentioned, some of these facilities request a local identification card for care (though respondents indicated that this practice may have decreased during the time of data collection). Some workers reported dissatisfaction (including poor reception, lack of appropriate medical examination and long waiting time) with the service provision at the public health facilities resulting in them visiting private clinics which cost more money. Some workers reported that they do not seek care when suffering from malaria symptoms due to these reasons. One worker talked about his encounter in a health centre as follows:

*“…I am sick today. I have been to the health centre. I was diagnosed with malaria. I was given anti-pain for the headache. They told me they run out of the right anti-malarial and informed me to buy from a private pharmacy. At the moment, I couldn’t afford to buy from a private pharmacy. And none of my colleagues have a tablet at hand. I am left with no options.’’*

Health workers said that they do occasionally conduct fever treatment campaigns at farms which employ migrant workers. Health workers also admitted that they do not consider the influx and increased number of migrant workers during the planning of malaria control efforts and the calculation of commodities needed. One farm was reported to cover the medical costs that were accrued during a worker’s malaria treatment, but this was not common.

## Discussion

Findings from this study indicate poor housing and living conditions for migrant workers in the study area and describe their increased risk for mosquito bites and malaria infection. They sleep in shelters with open eaves and doors allowing easy access for vector mosquitoes. Some workers occasionally sleep outdoors when the shelters are full or during the hot season for comfort, which again increases their risk for malaria infection. Only a small proportion of the workers used personal protection interventions to protect from mosquitoes because of the lack of access to interventions such as LLINs [2, 28] with few workers using any prevention methods to protect against mosquito bites during mosquito biting times (19:00–05:00).

Observation of all workers within the shelter revealed that most workers were indoors by 22:00, though as discussed, being inside the shelter provides only limited protection from mosquitoes. The study team also noted worker misconceptions about malaria prevention and treatment, illustrating the need for malaria prevention messages targeting the specific needs of migrant workers. The results of this study indicate that migrant workers carry a significant risk for mosquito bites in an area with high malaria burden which upon their return to their home communities could potentially facilitate the importation of malaria to areas with a lower malaria burden. Because of this potential movement of malaria across wide geographical areas, malaria in migrant workers can impact ongoing efforts to control and eliminate malaria throughout Ethiopia.

All agricultural activities in the selected farms were aligned with rainy season due to farms needing labour for preparation of the fields, planting, weeding and harvesting. These activities started between April and June depending on the type of plantation and size of farm. Harvesting was the most labour-intensive activity and required the largest number of migrant workers. The majority of the migrant workers arrived in June and returned to their home in December, which usually marks the end of the farming season. Migrant workers are at the farms during and after the rainy season, which corresponds with intense malaria transmission [2, 29], and move from lower malaria burden areas (their permanent residence) in search of work.

The roof of most shelters was made from grass and walls were made from grass or other materials. Most shelters had open eaves with no doors, or if doors were present they remained open at night, and none of the shelters had screens on the windows or eaves. Other studies describe similar living and housing condition of migrant workers [30]. In addition, the shelter characteristics observed could make LLINs impractical to hang due to the structure design with no individual sleeping spaces in a crowded multi-person shelter. Indoor residual spraying (IRS) would also have minimal effectiveness for such shelters due to the type of building materials and large gaps in the walls. Poor housing conditions in the farms increase the risk for mosquito bites and subsequent malaria infection. Increased risk of malaria infection as a result of poor housing and living conditions has been described elsewhere [31–33].

Sixty-nine percent of the shelters have at least one net. While this is similar to country-wide household net ownership reported by the MIS 2015 (64%) [2], worker shelters often contain many more people than an average household. The low LLIN coverage for individual migrant workers does not meet the World Health Organization recommendation of one net for every two individuals [34], and insights from the qualitative inquiry indicate the administrative and financial challenges of providing LLINs at the individual level. There was an obvious lack of LLINs available for migrant workers, which could be a component of future action. The problem is compounded by the continually changing worker population on farms as the monthly average of workers per shelter ranged from 6 to 30 on the farms in the study area, with much higher numbers of workers reported in shelters at nearby farms. The bed nets that were available to migrant workers were mostly old and torn which would decrease effectiveness and could have a cumulative effect on the vulnerability of migrant workers to malaria infection.

A major objective of this study was to describe bed net use and sleeping patterns. Overall, less than 25% of the migrant workers were sleeping under a net during the 4 observation months. This is below the use rates reported in the MIS 2015 which showed 45% of the general population slept under bed net the previous night nationwide [2]. The findings from other recent studies in several locations of the country showed use of bed nets ranging from 9 to 85% [30, 35] with the lowest being reported among migrant workers. None of those studies utilized direct observation of individuals, so utilization rates in other studies are based on the response of household or temporary shelter members (in case of migrant workers) about use of LLINs during the previous night rather than direct observation. As per respondent comments during interviews and discussions, the lack of access was the primary factor for low utilization rates. In addition to the low utilization, a significant number of migrant workers sleep outdoors or work during the night without any personal protection, also increasing their risk for mosquito bites [36–38].

Research conducted by Dugassa et al*.* (associated manuscript) was conducted simultaneously in the same location and workplaces to assess biting rates using human landing collection of mosquitoes at indoor and outdoor locations. As per this study, the aggregate (sum of indoor and outdoor) mosquito biting had a bimodal peak which was at 21:00 to 22:00 and 00:00 to 02:00. The mosquitoes were most aggressive from 00:00 to 02:00. Unfortunately, less than a quarter of the migrant workers were underneath bed nets during these hours in all 4 months. As pointed out, migrant workers were also not using any other alternative personal protection against mosquitoes during these hours. This indicates that mosquitoes had almost unrestricted access to feed on those migrant workers with no protection. The research by Dugassa et al*.* also demonstrated no significant difference between indoor and outdoor biting over the course of nighttime observations, illustrating the risk for workers both inside their shelter and those working or sleeping outdoors. There was a difference in biting times though as mosquitoes showed increased biting indoors from 20:00 to 21:00 and outdoors from 03:00 to 04:00. A large proportion of migrant workers were indoors from 20:00 to 21:00. In addition, close to 20% of migrant workers were outdoors from 03:00 to 04:00 in November and December. This information suggests that future interventions should address both indoor and outdoor worker exposures.

This study highlighted worker misconceptions about the cause of malaria and the role of mosquitoes in malaria transmission among migrant workers. A similar misperception about the cause of malaria was demonstrated by a community survey in Ethiopia [39]. However, the role of mosquitoes was correctly described by participants in other similar studies [39, 40]. The misconception among the migrant workers could be due to their low access to health extension services which provide health education information. This study did indicate a moderate level of awareness about the need to prevent malaria among the migrant workers. Migrant workers were able to discuss important malaria prevention activities, although they were unable to apply them due to lack of access and poor coordination among the farm management. Almost all the migrant workers described environmental management, use of LLINs and IRS to prevent malaria. This is consistent with other studies conducted in Ethiopia [39, 40]. Improving access of migrant workers to LLINs, malaria diagnosis, and malaria treatment, could be a future focus for public health action.

Due to low access to malaria prevention interventions, workers rely on prompt treatment when they have fever or malaria which can be challenging due to the absence of a fully functional clinic in the farms. This study indicated low access to malaria treatment for migrant workers. One of the contributing factors is the absence of active clinics on farms. Migrant workers were required to travel to public health facilities up to 30 km distant when they get sick. At the public health facilities, possible drug-stock outs may prevent them from obtaining anti-malarial drugs. It was also reported that migrant workers were not given treatment in public health facilities operating in the district due to the prioritization of malaria treatment for local community members. Rural communities in Ethiopia have designated health extension workers who diagnose and treat malaria though migrant worker shelter and workplaces are not often covered by health extension workers. Due to challenges in obtaining proper treatment, migrant workers were sometimes forced to accept poor treatment, which based on descriptions by respondents of this study, included incomplete treatments and sharing anti-malarial tablets. Proper documentation of drug adherence and the implications of poor adherence to emergence of drug resistance is an area for future research.

This is the first study to monitor worker behaviour by employing nighttime observations to explore behavioural risks for mosquito bites in the Ethiopian agricultural setting. Conducting direct observations of migrant workers provided an opportunity to compile data about worker activities, perspectives, and actual living conditions and behaviours to identify unique needs among migrant worker populations for health outreach, public private partnerships with farm ownership, and malaria policy. Use of combined methods allowed triangulation and reverification of the data which provided robust findings. In addition, the study accommodated the participation of critical stakeholders, such as migrant workers, farm management, and health providers to include varying opinions and perspectives. Collecting the study data from only one district and eight farms does represent a limited number of the total farms, and their selection was not random but was guided by logistical considerations, but the inclusion of different farm types was designed to capture different farm workplace settings. Future investigations will hopefully build upon this study’s findings and include more locations and farms throughout Ethiopia.

## Conclusion and recommendation

Migrant workers arrived to work at the farms during the peak malaria transmission season. They suffered from repeated malaria illness during their stay at the farms, and while there, lacked access to prevention and treatment. Migrant workers living in highland areas with lower burdens of malaria then contribute to malaria transmission when they return to their home communities.

The following recommendations could be considered for improved malaria control among the migrant workers and the nation as a whole. All stakeholders, including migrant workers, farm managers, farm owners, public health officials and government leaders should collaborate to develop a comprehensive approach and partnership to address malaria prevention and treatment among migrant worker populations. Given the complexity of the problem, any future intervention could begin with strategic planning, defining migrant worker populations, describing a population movement framework, and developing specific government policies to target migrant workers and their workplaces.

Future interventions can focus on providing optimal care to migrant workers using the existing public health infrastructure, such as the implementation of mobile health clinics to address migrant worker populations. However, this may require increased health resources and technical support for districts which accommodate large migrant populations. Public–private partnerships could be initiated with farms that employ migrant workers, especially those located a long distance from a public health facility, to support private farm clinics or training farm employees to provide necessary malaria testing and treatment. Increased engagement with farm owners about the benefits of providing malaria treatment onsite, providing LLINs to workers, advantages of properly constructed structures, and the value of a healthy workforce would be helpful as well as strengthening referral linkages with public facilities. Regular IRS treatment campaigns by the district health professionals would also be beneficial. Improvement of housing through construction of sprayable walls would allow IRS to be conducted in these structures, and screening of the structures could reduce exposure to mosquitoes by workers not using LLINs. Increased access to LLINs is an obvious need and scale up of this intervention could be conducted by individual farms, or through increased provision of LLINs at local health clinics. Additionally, there is a clear need to explore alternative malaria prevention measures which are feasible for both indoor and outdoor settings, such as spatial repellents or placement of worker structures in areas away from larval breeding sites.

Service delivery to migrant workers could be integrated with the residential community and addressed using mobile health clinics. Health extension workers can address many of the diagnosis and treatment issues faced by migrant workers, but that would be a significant burden on health extension workers and other public health officials. A sustainable, long-term approach to address migrant worker malaria treatment and care could be developed with all levels of government administration.

The supply chain for malaria commodities, such as bed net and anti-malarial planning and distribution, remains an opportunity for improvement. To inform these decisions, the seasonal influx of migrant workers could be calculated every year. This could be accomplished by having farms register their workers and have health clinics collect data about the number of migrant workers they treat each year. Alternatively, a “travelling net” could be provided to migrant workers before they leave for temporary work on farms. Migrant workers are critical to a healthy economy in Ethiopia and lessons learned from this study may help guide future malaria prevention and elimination policy and decision-making in Ethiopia.

## Data Availability

The dataset is available on request from Dr. Asfawesen Gebreyohannes, Deputy Chief of Party to PHSP which is managed by Abt Associates Inc, Ethiopia office. E-mail: asfawesengy@phsp-et.com.

## Abbreviations

LLINs: Long-lasting insecticidal nets
FGDs: Focus group discussions
SSIs: Semi-structured interviews
